# Validity and reliability of the Korean version of the pediatric quality of life ESRD module

**DOI:** 10.1186/1477-7525-10-59

**Published:** 2012-06-06

**Authors:** Ki-Soo Park, Min Hyun Cho, Il Soo Ha, Hee Gyung Kang, Hae Il Cheong, Young Seo Park, Yoon Jung Lee, Joo Hoon Lee, Hee Yeon Cho

**Affiliations:** 1 Department of Preventive Medicine and Institute of Health Sciences, Gyeongsang National University Hospital, 816-15 Jinjudae-ro, Jinju, 660-751, South Korea; 2 Department of Pediatrics, Kyungpook National University Hospital, 130 Dongduk-ro, Jung-gu, Daegu, 700-721, South Korea; 3 Department of Pediatrics, Seoul National University Children’s Hospital, 101 Daehak-ro, Jongno-gu, Seoul, 110-744, South Korea; 4 Department of Pediatrics, Asan Medical Center, 88, Olympic-ro 43-gil, Songpa-gu, Seoul, 138-736, South Korea; 5 Department of Pediatrics, Samsung Medical Center, 50 Irwon-dong, Gangnam-gu, Seoul, 130-710, South Korea

**Keywords:** Quality of life, Children, End-stage renal disease

## Abstract

**Background:**

Health-related quality of life is a very important issue in children with end-stage renal disease and their family. Moreover, this can be a lifelong problem. In this study, we performed a cross-sectional investigation of the health-related quality of life in Korean children, undergoing renal replacement therapies, such as dialysis and renal transplantation.

**Findings:**

We validated the Korean version of the PedsQL 3.0 End-Stage Renal Disease Module by comparing with the PedsQL 4.0 Generic Core Scales. A total of 92 pediatric patients with end-stage renal disease, aged 2–18 year old, were enrolled in four teaching hospitals in Korea. The module was acceptable for both parent proxy-report and child self-report. The response rate was acceptable, since no reminders were delivered. A large proportion of the responders answered >90% of the items, which suggests a good face validity. The PedsQL 4.0 Generic Core Scales and the PedsQL 3.0 End-Stage Renal Disease Module showed minimal missing values in the current study, which supported feasibility. The validation analyses revealed acceptable floor and ceiling effects and an acceptable construct validity.

**Conclusions:**

The PedsQL 3.0 End-stage Renal Disease Module may be useful as an end-stage renal disease -specific instrument in the evaluation of the health-related quality of life in Korean children; however, a larger, longitudinal prospective study is needed.

## Findings

### Introduction

Recent medical advances have resulted in remarkable improvements in the treatment of patients with chronic diseases, especially, in children [1-3]. However, end-stage renal disease (ESRD) practically affects every organ system, and thus, has a major impact, not only on mortality, but also on the quality of life (QOL) of the children suffering from the disease [4]. Systemic assessment of the health-related quality of life (HRQOL) in these children must be another essential process that may help promote a pediatric patient’s successful transition into adulthood [5].

Recently, researchers have used pediatric HRQOL measures as a tool to evaluate healthcare services in an effort to improve the patient health and their well-being [6,7]. For pediatric patients with ESRD, there has been an increasing clinical interest in their QOL, and their psychosocial adaptation [3,5,8-16].

To measure HRQOL, among the children with ESRD, we used several instruments, including the Pediatric Quality of Life Inventory™ (PedsQL™) Generic Core Scales [17], which contain general domains, but no ESRD-specific domains. Because of the need for a more appropriate measurement, the PedsQL™ End Stage Renal Disease Module [3] was developed as a PedsQL™ disease-specific module. The PedsQL™ was designed under a modular-approach [18] to cover both the generic and disease-specific domains. The PedsQL™ End Stage Renal Disease Module was developed through the focus groups, involving the healthcare providers, children, and parents; cognitive interviews; pre-testing; and field-testing [3].

The goal of this study is to assess the psychometric properties of the Korean translation of the PedsQL™ End Stage Renal Disease.

## Methods

### Participants and settings

We performed a cross-sectional study of the Korean translations of the PedsQL End Stage Renal Disease Module, comparing the child self-reported and parent-proxy–reported HRQOL of the children with ESRD. The PedsQL 3.0 End Stage Renal Disease Module was administered from January 2011 through June 2011, one time each to the child and parent at four pediatric ESRD centers. Participants included children aged 2 to 18 years that received maintenance dialysis treatment or renal transplant care for at least 6 months and their parents. Child self-report includes ages 5–7, 8–12, and 13–18. Parent proxy-report includes ages 2–4 (toddler), 5–7 (young child), 8–12 (child), 13–18 (adolescent), and assesses the parent’s perception of their child's HRQOL.

The human subject institutional review boards, at each institution, approved the study. Written parental informed consent and child assent were obtained before the enrollment of the subjects.

### Measures

#### Translation - The Korean translations of the Pediatric Quality of Life Inventory™ (PedsQL™ 3.0) Version 3.0 End Stage Renal Disease Module

After we acquired permission from the original developer (Mapi Research Trust, on behalf of Dr. James W. Varni, copyright owner in the PedsQL™ ) for the original PedsQL 3.0 End Stage Renal Disease Module, the original English version of the PedsQL 3.0 End Stage Renal Disease Module was first translated into Korean, by a native Korean professional translator, who majored in English literature. Another bilingual translator backward-translated this revised Korean version of the PedsQL 3.0 End Stage Renal Disease Module into English. The correspondence between this back-translated version and the original English version was reviewed by the panel again. After considering the original, translated, and back-translated versions all together, we produced a pilot Korean version of the PedsQL 3.0 End Stage Renal Disease Module. Five children, from each age group participated in the pilot testing along with their parents. A researcher (MH or KS) measured the time taken to complete the questionnaire. Upon completing the questionnaire, the researcher interviewed each child and his or her parent, and the thought processes used in answering the questionnaire were deduced by cognitive interviewing [19]. A final version of the Korean version of the PedsQL™ 3.0 End Stage Renal Disease Module was produced after revising the pilot version using data obtained during pilot testing.

### Measurements

Parents completed a modified PedsQL 4.0 Generic Core Scales, PedsQL 3.0 End Stage Renal Disease Module that contained demographic information, including the child's date of birth, sex, parental age and education level, and patient's diagnosis age and duration.

#### PedsQL 3.0 end stage renal disease module

The 34-item PedsQL 3.0 End Stage Renal Disease Module includes 7 scales. The scales are composed of both the child self-report and parent-proxy report formats for children aged 5 to 18 years, and a parent-proxy report format for children aged 2 to 4 years. The format, instructions, Likert response scale, and scoring method were identical to the PedsQL 4.0 Generic Core Scales, with higher scores indicating better HRQOL [5].

### Statistical analysis

The psychometric elements of the PedsQL 3.0 End Stage Renal Disease Module were examined in two parts: First, we assessed the data quality and internal consistency. Second, we compared PedsQL 3.0 End Stage Renal Disease Module and PedsQL 4.0 Generic Core Scales. Analyses were performed with SPSS 15.0 (SPSS, Chicago, IL).

Data quality was assessed by the mean, extent of ceiling and floor effects. Floor and ceiling effects between 1% and 15% were defined as optimal [20]. Internal consistency has been assessed with the use of Cronbach’s alpha. While tests for construct validity usually compared PedsQL 4.0 Generic Core Scales, a spearman rank correlation test was used as a nonparametric approach [3], which is also used in most validation studies. Agreement between the child self-report and parent proxy-report was determined, through a two-way mixed effect model (absolute agreement, single measure) intraclass Correlation Coefficients (ICC) [3]. The ICC offers an index of absolute agreement, given that it takes into account the ratio between the subject variability and total variability [3,21,22]. ICCs are designated as ≤ 0.40 poor to fair agreement, 0.41–0.60 moderate agreement, 0.61–0.80 good agreement, and 0.81–1.00 excellent agreement [3].

## Results

A total of 92 children and adolescents (aged 2 to 18 years) with ESRD and their parents were enrolled in our study. Both the child self-report and parent-proxy report were available for the eighty six children aged 5 to 18 years. Most of the parent proxy reports in our study were completed by the mothers except one. The sample consisted of children receiving hemodialysis (n = 11; 12.0%), peritoneal dialysis (n = 44; 47.8%) and a renal transplant (n = 37; 40.2%) (Table 1).

**Table 1:** Patients Characteristics

	**Total No.(n,%)**	**Total**		**Hemodialysis**		**Peritoneal Dialysis**		**Transplant**	
		**N**	**%**	**N**	**%**	**N**	**%**	**N**	**%**
	**Gender**								
	Male	44	47.8	5	11.4	20	45.5	19	43.2
	Female	48	52.2	6	12.5	24	50.0	18	37.5
	**Age (mean ± SD)**	12.1 ± 4.21		11.6 ± 5.26		12.6 ± 4.25		11.6 ± 3.84	
	2-4 years: toddler	6	6.5	2	33.3	3	50.0	1	16.7
	5-7 years: young child	8	8.7	1	12.5	3	37.5	4	50.0
	8-12 years: child	26	28.3	1	3.8	10	38.5	15	57.7
	13-18 years: adolescent	52	56.5	7	13.5	28	53.8	17	32.7
	**Cause of ESRD**								
Child	CGN	37	40.2	4	10.8	21	56.8	12	32.4
	CPN	3	3.3	0	0.0	1	33.3	2	66.7
	Genetic Disease	18	19.6	2	11.1	7	38.9	9	50.0
	Others	20	21.7	5	25.0	9	45.0	6	30.0
	Unknown cause	14	15.2	0	0.0	6	42.9	8	57.1
	**Age at diagnosis of ESRD**								
	≤ 4 years	28	30.4	5	17.9	7	25.0	16	57.1
	> 4 years	64	69.6	6	9.4	37	57.8	21	32.8
	**Duration after diagnosis of ESRD**								
	< 5 years	51	55.4	6	11.8	35	68.6	10	19.6
	≥ 5 years	41	44.6	5	12.2	9	22.0	27	65.9
	**Father's age (mean ± SD)**	45.2 ± 5.03		44.3 ± 4.43		45.7 ± 5.18		44.8 ± 5.09	
	< 40 years	14	15.2	2	14.3	5	35.7	7	50.0
	≥ 40 years	78	84.8	9	11.5	39	50.0	30	38.5
	**Father's educational level**								
	High school or below	44	47.8	5	11.4	19	43.2	20	45.5
	College or over	48	52.2	6	12.5	25	52.1	17	35.4
	**Mother's age (mean ± SD)**	42.5 ± 4.77		40.8 ± 4.14		43.3 ± 5.18		41.9 ± 4.28	
Parent	< 40 years	26	28.3	5	19.2	8	30.8	13	50.0
	≥ 40 years	66	71.7	6	9.1	36	54.5	24	36.4
	**Mother's educational level**								
	High school or below	53	57.6	7	13.2	25	47.2	21	39.6
	College or over	39	42.4	4	10.3	19	48.7	16	41.0
	**Parental marital status**								
	Married	82	89.1	9	11.0	41	50.0	32	39.0
	Divorced, Separated	10	10.9	2	20.0	3	30.0	5	50.0
	**Total**	92	100.0	11	12.0	44	47.8	37	40.2

CGN, chronic glomerulonephritis; CPN, chronic pyelonephritis; ESRD, end-stage renal disease.

The percentage of missing item responses was less than 1.1% for the child self-report and parent proxy report. The average scores of the parent proxy report were 45.4-72.3 and the child self-report were 57.5-71.4. Ceiling effects occurred between 1.1% and 14.1% and floor effects occurred between 4.3% and 14.1%; thus, these effects did not represent a limitation of the PedsQL End Stage Renal Disease Module.

Internal consistency reliability coefficients for the PedsQL 3.0 End Stage Renal Disease Module are listed in Table 2. All parent-proxy report scales, except for one, exceeded the minimum reliability standard of 0.70, and four child self-report scales exceeded the minimum reliability standard.

**Table 2:** Scale Descriptives for the PedsQL™ 3.0 ESRD module

	**Mean**	**SD**	**Ceiling**	**Floor**	**Cronbach's alpha**
			**Effects (%)**	**Effects (%)**	
**Parent-proxy report**					
General Fatigue	47.0	22.31	3.3	2.2	0.88
About My Kidney Disease	62.8	19.60	5.4	1.1	0.83
Treatment Problems	65.0	18.55	3.3	1.1	0.65
Family and Peer Interaction	52.5	22.76	5.4	4.3	0.81
Worry	45.4	22.83	1.1	3.3	0.92
Perceived Physical Appearance	51.5	24.74	6.5	4.3	0.70
Communication	72.3	19.70	14.1	1.1	0.91
**Child-self report**					
General Fatigue	57.5	21.22	4.3	1.1	0.83
About My Kidney Disease	67.7	18.57	6,5	2.2	0.77
Treatment Problems	67.2	18.50	7,6	1.1	0.57
Family and Peer Interaction	65.1	22.24	12.0	1.1	0.70
Worry	63.2	20.77	5.4	2.2	0.90
Perceived Physical Appearance	59.5	25.46	12.0	2.2	0.68
Communication	71.4	21.19	14.1	1.1	0.87

Intercorrelations, between the Generic Core Scales and summary scores with the PedsQL 3.0 End Stage Renal Disease Module, are listed in Table 3. The majority of intercorrelations were in the medium to large range, supporting the construct validity.

**Table 3:** Correlation Coefficients among PedsQL Scales for the Child Self-Report and Parent-Proxy Report for the ESRD Sample

		**Child Self-Report**							**Parent-Proxy Report**						
	**GF**	**KD**	**TP**	**F/PI**	**W**	**PA**	**C**	**GF**	**KD**	**TP**	**F/PI**	**W**	**PA**	**C**	
Child														
GF	1													
KD	0.45^**^	1												
TP	0.18	0.27^**^	1											
F/PI	0.40^**^	0.29^**^	0.56^**^	1										
W	0.36^**^	0.41^**^	0.38^**^	0.63^**^	1									
PA	0.30^**^	0.61^**^	0.40^**^	0.49^**^	0.52^**^	1								
C	0.36^**^	0.28^**^	0.24^*^	0.48^**^	0.34^**^	0.38^**^	1							
Parent's														
GF	0.57^**^	0.38^**^	0.31^**^	0.47^**^	0.36^**^	0.36^**^	0.31^**^	1						
KD	0.36^**^	0.54^**^	0.36^**^	0.41^**^	0.35^**^	0.38^**^	0.21^*^	0.61^**^	1					
TP	0.19	0.23^*^	0.60^**^	0.42^**^	0.24^*^	0.32^**^	0.14	0.43^**^	0.56^**^	1				
F/PI	0.51^**^	0.19	0.32^**^	0.53^**^	0.44^**^	0.25^*^	0.32^**^	0.63^**^	0.53^**^	0.44^**^	1			
W	0.34^**^	0.21^*^	0.21^*^	0.34^**^	0.41^**^	0.152	0.24^*^	0.42^**^	0.47^**^	0.41^**^	0.56^**^	1		
PA	0.40^**^	0.36^**^	0.37^**^	0.38^**^	0.38^**^	0.51^**^	0.27^*^	0.54^**^	0.60^**^	0.51^**^	0.63^**^	0.51^**^	1	
C	0.35^**^	0.28^**^	0.24^*^	0.38^**^	0.34^**^	0.32^**^	0.52^**^	0.44^**^	0.37^**^	0.24^*^	0.42^**^	0.36^**^	0.41^**^	1
Child														
PH	0.51^**^	0.50^**^	0.28^*^	0.53^**^	0.47^**^	0.49^**^	0.34^**^	0.46^**^	0.42^**^	0.21	0.35^**^	0.29^**^	0.33^**^	0.34^**^
PsyH	0.51^**^	0.45^**^	0.48^**^	0.64^**^	0.58^**^	0.48^**^	0.48^**^	0.50^**^	0.45^**^	0.42^**^	0.47^**^	0.41^**^	0.40^**^	0.47^**^
EF	0.42^**^	0.45^**^	0.49^**^	0.59^**^	0.61^**^	0.54^**^	0.41^**^	0.44^**^	0.44^**^	0.45^**^	0.32^**^	0.37^**^	0.41^**^	0.45^**^
SF	0.44^**^	0.40^**^	0.40^**^	0.59^**^	0.49^**^	0.48^**^	0.40^**^	0.42^**^	0.41^**^	0.35^**^	0.39^**^	0.26^*^	0.32^**^	0.41^**^
SchH	0.44^**^	0.28^**^	0.33^**^	0.44^**^	0.38^**^	0.20	0.40^**^	0.41^**^	0.30^**^	0.28^**^	0.48^**^	0.40^**^	0.27^*^	0.34^**^
Parent's														
PH	0.34^**^	0.31^**^	0.23^*^	0.31^**^	0.29^**^	0.23^*^	0.10	0.53^**^	0.58^**^	0.29^**^	0.47^**^	0.34^**^	0.49^**^	0.34^**^
PsyH	0.49^**^	0.29^**^	0.31^**^	0.42^**^	0.36^**^	0.28^**^	0.32^**^	0.57^**^	0.63^**^	0.37^**^	0.56^**^	0.44^**^	0.51^**^	0.46^**^
EF	0.38^**^	0.36^**^	0.35^**^	0.37^**^	0.46^**^	0.33^**^	0.29^**^	0.53^**^	0.61^**^	0.35^**^	0.42^**^	0.37^**^	0.49^**^	0.43^**^
SF	0.44^**^	0.15	0.22^*^	0.34^**^	0.19	0.24^*^	0.24^*^	0.48^**^	0.53^**^	0.32^**^	0.52^**^	0.30^**^	0.45^**^	0.36^**^
SchH	0.45^**^	0.24^*^	0.23^*^	0.37^**^	0.29^**^	0.15	0.30^**^	0.45^**^	0.48^**^	0.30^**^	0.49^**^	0.46^**^	0.38^**^	0.39^**^

Abbreviations: *GF*, general fatigue; *HD*, about my kidney disease; *TP*, treatment problems; *F/PI*, family and peer interaction; *W*, worry; *PA*, perceived physical appearance; *C*, communication; *PH*, physical functioning; *PsyH*, psychosocial functioning; *EF*, emotional functioning; *SF*, social functioning; *SchH*, school functioning.

*p < 0.05, **p < 0.01.

Table 4 lists ICCs between the children self-report and parent proxy report. ICCs are in the moderate or good agreement range for 11 of 12 PedsQL Scales and in the poor agreement range for 1 of 12 PedsQL Scales. The greatest overall agreement is found on the School function (0.73) and the lowest agreement is found on the Worry (0.35).

**Table 4:** ICCs Between Child Self-Report and Parent-Proxy Report for the PedsQL 4.0 Generic Core Scales and PedsQL 3.0 ESRD Module for ESRD Sample

**Scale**	**ICC**
Total ESRD score	0.57^**^
General Fatigue	0.49^**^
About My Kidney Disease	0.52^**^
Treatment Problems	0.60^**^
Family and Peer Interaction	0.42^**^
Worry	0.35^*^
Perceived Physical Appearance	0.48^**^
Communication	0.52^**^
Physical Health	0.44^**^
Psychosocial Health	0.58^**^
Emotional Functioning	0.49^**^
Social Functioning	0.45^**^
School Functioning	0.73^**^

*p < 0.01, **p < 0.001.

## Discussion

We have translated and adapted the original 34-items of PedsQL 3.0 End Stage Renal Disease Module into a Korean version. The Korean, as well as the English version of the PedsQL 3.0 End Stage Renal Disease Module, represents a comprehensive, brief and low time consuming instrument to assess the quality of care of pediatric ESRD patients.

According to the recent Consensus-based Standards (COSMIN) of assessing the methodological quality of patient reported measures [23], reliability, validity and responsiveness must be evaluated before these instruments can be used for research and development. Thus, we needed to first look at the content, criteria and construct validity of the PedsQL 3.0 End Stage Renal Disease Module, since this study may point out critical issues.

The response rate was acceptable since no reminders were delivered. A large proportion of the responders answered >90% of theitems, suggesting good face validity. In addition, the PedsQL 4.0 Generic Core Scales and the PedsQL 3.0 End Stage Renal Disease Module showed minimal missing values in the current study, supporting feasibility [3]. The validation analyses revealed acceptable floor and ceiling effects, and an acceptable internal consistency for the seven proposed subscales and a reasonable good model fit of the proposed factorial structures.

Our findings showed that the instrument had reasonably good internal’s reliability, among the ESRD patients; the results of the assessment of internal consistency showed that the PedsQL 3.0 End Stage Renal Disease Module appeared to measure what it is supposed to. For the PedsQL 3.0 End Stage Renal Disease Module, six of seven parent-proxy report scales, and five of seven child self-report scales exceeded the minimum coefficient standard of 0.70. Scales that met the minimum coefficient standard of 0.70 may be used to examine the specific domains of HRQOL, as well as subgroup differences. In the current study, only the Cronbach’s alpha for the Treatment Problems Scale for child self-report was problematic, which is similar to the original US study [3]. As noted by Goldstein et al. [3], the Treatment Problems Scale is not conceptually an HRQOL scale as such, but rather a treatment barriers scale, and in the adult patients, higher perceived barrier scores were significantly associated with lower self-reported adherence and poorer prescription refill rates. However, parents provided more prompts to their children to take medication if their children had a greater number of problems [24]. That is, a remembrance to take medications that is an item of Treatment Problems Scale is affected by their parents in Korea, the other three treatments barrier items are affected by their self. The other child self-report scales are over or near 0.70. These items were shown to be selective and non-redundant, which resulted in very satisfactory results for Cronbach’s alpha [25].

In this study, children with ESRD and their parents were shown to have poor to good agreement at most. These results indicate that the information provided by proxy respondents is not always equivalent to that reported by the patient. Child age and parental distress can affect the children’s and parents’ perceptions of the health and well-being of the child [3].

The present study has several limitations. Although our recruitment strategy enabled us to generalize beyond a clinical sample, and was comparable to the other samples, in terms of age and sex, the sample was a purposive one. Further, information for nonparticipants was not available, and in addition, the sample size for young children with ESRD was small and the sample size of older children was large, which may limit generalizability. Although the number of patients used in this study was lower (92 patients), these enrolled patients consisted of about 31% of the total pediatric ESRD patients in our country, the so called ‘The Korea Pediatric CKD registry’, which has been operated from 2007 to 2010. A total of 294 patients (hemodialysis: 27 patients, peritoneal dialysis: 99 patients, renal transplantation: 168) were registered to that registry. At present, the most common causes of ESRD in adults are diabetes mellitus (DM) and hypertension, but, in children, glomerulonephritis and chronic pyelonephritis are most common causes, similar to that of the previous reports. Although the prevalence rate of DM and hypertension in children are markedly increasing, these causes cannot develop ESRD within the childhood period because of long duration for progression to ESRD. Therefore, the incident rate of ESRD in children is extremely rare (only 4 children/million), and adolescent patients tend to be relatively more than young children during childhood period.

This study demonstrated that the PedsQL 3.0 End Stage Renal Disease Module was a feasible, reliable, and a valid instrument to assess the specific HRQOL in children with ESRD, and the PedsQL 3.0 End Stage Renal Disease Module represented a realistic and relevant landscape of the components relevant for the future evaluation of Korean chronic care.

## Abbreviations

C: Communication; CFI: Comparative Fit Index; CGN: Chronic GlomeruloNephritis; CPN: Chronic PyeloNephritis; EF: Emotional Functioning; ESRD: End-Stage Renal Disease; F/PI: Family and Peer Interaction; GF: General fatigue; HD: About my kidney disease; HRQOL: Health-related Quality of Life; PA: Perceived Physical Appearance; PedsQL™: Pediatric Quality of Life Inventory™; PH: Physical Functioning; PsyH: Psychosocial Functioning; RMSEA: Root Mean Square Error of Approximation; SchH: School Functioning; SF: Social Functioning; TP: Treatment Problems; W: Worry; WLSMV: Weighted Least-Squares Method.

## Competing interests

The authors declare that they have no competing interests.

## Authors’ contributions

KS, MH were the main investigator, participated in the collection of data, design of the study, analysis and drafted the manuscript. IS, HG, HI, YS, YJ, KH and HY have collected data and made interpretation of data, have been revising it critically for intellectual content. All authors read and approved the final manuscript collected the data.
